# Management of borderline ovarian tumors: A tertiary referral center experience in Egypt

**DOI:** 10.3389/fsurg.2022.962820

**Published:** 2022-09-02

**Authors:** Khaled Gaballa, Mohamed Abdelkhalek, Adel Fathi, Basel Refky, Khaled Belal, Moustafa elaraby, Mohammad Zuhdy

**Affiliations:** ^1^ Department of Surgical Oncology, Oncology Center, Mansoura University, Mansoura, Egypt; ^2^ Ministry of Health, Consultant of Obstetrics and Gynecology, Mansoura, Egypt

**Keywords:** borderline, ovarian borderline tumor (BOT), ovarian borderline malignancy, ovarian cancer, borderline (»proliferating«) mucinous tumor

## Abstract

**Background:**

In this retrospective study, we discuss our experience as a large tertiary referral center in Egypt in the management and follow-up of borderline tumors

**Patients and methods:**

This is a retrospective cohort study where all patients diagnosed with a borderline ovarian tumor at Oncology Center Mansoura University from November 2014 to June 2020 were included. Demographics, preoperative, operative, postoperative, pathologic, and oncologic follow-up data were retrieved from a prospectively maintained electronic database. The included patients were followed until April 2022.

**Results:**

We included 27 patients with borderline ovarian tumors. The mean age of the study patients was 47.67 ± 16.39 years. The median CA 125 was 33 (6–304 U/ml). Frozen section examination was utilized in 13 patients (48.14%), where a diagnosis of borderline ovarian tumors was revealed in 8 patients. Recurrence was reported in one patient with serous type after approximately 26 months. The most common pathological type in our cohort was the mucinous borderline type reported in 14 patients (51.9%), followed by the serous type reported in 11 patients (40.7%), and the seromucinous type in 1 patient only. Patients with mucinous borderline type were significantly younger (40.083 ± 18.47 vs. 53.73 ± 11.91 years, *p* = 0.028). Interestingly, Cancer Antigen 125 levels were significantly higher in mucinous than serous and seromucinous types [67(16–304) vs. 20(6–294.6) U/ml, *p* = 0.027]. On the other hand, the radiological tumor size of serous and seromucinous types was larger than that of the mucinous type [23(19–31) cm vs. 8(5–20) cm, *p* = 0.001]. Over a median follow-up period of 58.66 (54.16–63.16) months, only one postoperative mortality was reported, while only one recurrence was reported.

**Conclusion:**

Borderline ovarian tumors still represent a dilemma either in diagnosis or management. A frozen section examination could help to reach a preliminary diagnosis. Total abdominal hysterectomy and bilateral salpingo-oophorectomy are the cornerstone of surgical management; however, fertility-sparing surgery could be a valid option for women desiring fertility.

## Introduction

Borderline ovarian tumors (BOTs) are known to be a certain type of tumor that has a higher mitotic activity than the benign tumors but with no stromal invasion like other malignant epithelial ovarian tumors (1). Borderline ovarian tumors were first described in the literature about 100 years ago, and they nearly account for 10%–15% of all ovarian tumors (2, 3). Serous borderline tumors account for the majority of BOT with an incidence of about 50%, followed by mucinous type, which accounts for about 40% of diagnosed BOTs (4). BOTs are usually diagnosed during the reproductive age, with nearly 30% of cases diagnosed before 40 years (5, 6), and luckily, most of these tumors are diagnosed at an early stage while still confined to the ovaries with a 5-year survival rate approaching 95% (7). Radical surgery including hysterectomy and salpingo-oophorectomy had been the gold standard for the management of these tumors for decades; however, fertility-sparing surgery with preservation of the uterus and at least one ovary has been widely adopted recently as a better choice in the management of these tumors without compromising the oncological outcome, especially in the young age group of patients (8–11). Unilateral cystectomy also had been offered as a treatment option in the case of unilateral tumors; however, several studies have reported a higher recurrence rate despite not affecting the overall survival (8–11). The use of frozen sections in ovarian tumors has been increasing recently in several institutes worldwide; however, the diagnosis of BOT, particularly by frozen sections, is a real challenge with lower sensitivity and specificity rates than benign and malignant tumors (12–14).

Herein, we share and discuss our experience as a large tertiary referral center in Egypt in the management and follow-up of borderline tumors.

## Patients and methods

This is a retrospective cohort study where all patients diagnosed with a borderline ovarian tumor at Oncology Center Mansoura University from November 2014 to June 2020 were included. We included all patients who had a pathological diagnosis of borderline ovarian neoplasm, either the serous, mucinous, or seromucinous type. Patients who were operated by either laparotomy or laparoscopy, fertility-preserving surgery, or total abdominal hysterectomy and bilateral salpingoophrectomy were included. Patients diagnosed with invasive epithelial carcinoma or those with missing data were excluded. Demographics, preoperative, operative, postoperative, pathologic, and oncologic follow-up data were retrieved from a prospectively maintained electronic database. The included patients were followed until April 2022.

Data were fed to the computer and analyzed using IBM Corp. Released 2013 (IBM SPSS Statistics for Windows, Version 22.0; IBM Corp., Armonk, NY). Qualitative data were described using numbers and percentages. Quantitative data were described using median (minimum and maximum) and mean ± standard deviation for parametric data after testing normality using the Kolmogorov–Smirnov test. The significance of the obtained results was judged at the 0.05 level. The chi-square test, Fischer exact test, and Monte Carlo test for comparison of two or more groups as appropriate were used for qualitative variables. Student’s *t*-test and the Mann–Whitney *U* test were used to compare two independent groups of normally and non-normally distributed data.

## Results

We included 27 patients with borderline ovarian tumors. The mean age of the study patients was (mean ± standard deviation 47.67 ± 16.39 years). Most of the patients (86.4%) presented with vague abdominal symptoms and abdominal mass. The median CA 125 was 33 (6–304 U/ml). The remaining demographic and preoperative criteria are listed in Table 1.

**Table 1:** Sociodemographic characteristics and radiological data of the studied cases.

Age in years, mean ± SD (min–max)	47.67 ± 16.39 (17–76)	
Body mass index (kg/m^2^)	30.70 ± 7.32	
mean ± SD (min–max)	18.6–44.7	
Most common presentation		
Pain	10	45.5
Mass	9	40.9
Gastrointestinal symptoms	1	4.5
Abnormal uterine bleeding	1	4.5
Preoperative morbidity		
No	18	63.0
Diabetes	2	7.4
Hypertension and DM	3	11.1
Hypertension	2	7.4
Asthma	1	7.4
Stroke	1	3.7
CA 125 (U/ml)	33 (6–304) U/ml	
Ultrasonography		
Not performed	6	22.2
Performed	21	77.8
Computed tomography		
Not performed	10	37.0
Performed	17	63.0
Magnetic resonance imaging		
Not performed	12	44.4
Performed	15	55.6
Mass side in ultrasonography		
Unilateral	18	85.7
Bilateral	3	14.3
Mass size in ultrasonography, median (min–max) (cm)	14.5 (5.0–30.0)	
Mass side in computed tomography		
Bilateral	3	12.0
Left	8	32.0
Right	5	20.0
Mass side in MRI		
Right	8	50.0
Left	5	31.2
Bilateral	3	18.8
MRI mass size (cm)	17 (5–27)	

SD, standard deviation; min, minimum; max, Maximum; DM, diabetes mellitus; CA 125, Cancer Antigen 125; MRI, magnetic resonance imaging.

The tumor affected one ovary in 22 patients (81.4%), while it was bilateral in 5 patients (18.6%). Total abdominal hysterectomy and bilateral salpingo-oophorectomy were performed in 19 patients (70.3%), while unilateral oophorectomy was performed in 7 patients (25.7%). One patient had bilateral seromucinous borderline tumor and was managed by bilateral cystectomy only as fertility-preserving surgery after a multidisciplinary decision. The infracolic omentum was resected in 16 patients (59.25%) and was proved to be free from deposits after pathological evaluation. Lymphadenectomy was done in eight patients (32%), whereas pelvic lymphadenectomy was done in five patients, paraortic in one patient, and both lymph node groups in two patients. All dissected lymph nodes were free from tumor tissue. It should be noted that there was a clinical suspicion of ovarian cancer preoperatively in those patients.

The frozen section examination was utilized in 13 patients (48.14%), where a diagnosis of borderline ovarian tumors was revealed in 8 patients. The laparoscopic approach was used in two patients where conversion to open was performed in one of them due to adhesions. Table 2 illustrates the operative and postoperative parameters of the studied patients.

**Table 2:** Operative and postoperative parameters of the studied patients.

Parameter	No. of patients	Percentage
Surgery type		
Unilateral oophorectomy	7	25.7
TAH + BSO + omentectomy + lymphadenectomy	9	33.3
TAH + BSO + omentectomy	6	22.2
TAH + BSO	4	14.8
Bilateral ovarian cystectomy	1	3.7
Tumour side		
Right	11	40.7
Left	11	40.7
Bilateral	5	18.6
Operative time (minutes)	120 (45–330)	
Blood transfusion		
No	25	92.6
Yes	2	7.4
Intraoperative complication		
No	25	92.6
Yes	2	7.4
Intraoperative frozen section examination		
No	14	51.9
Yes	13	48.1
Intraoperative frozen section examination results	*n* = 13	
Borderline mucinous neoplasm with possible invasion	3	23.1
Cystic ovarian neoplasia	1	7.7
Borderline mucinous tumor with Brenner tumor	2	15.4
Mucinous cystadenoma	4	30.8
Borderline ovarian tumor	1	7.7
Seromucinous ovarian neoplasm, with free outer surface	1	7.7
Borderline serous tumor	1	7.7
Type of intraoperative complications	*N* = 2	7.4
Small intestinal injury	1	3.7
Urinary bladder injury	1	3.7
30-day postoperative complications		
Yes	4	14.81
Left hydronephrosis	1	
Burst abdomen	1	
Wound infection + pulmonary embolism	1	
Wound seroma	1	
Postoperative mortality	1	

TAH + BSO, Total abdominal hysterectomy and bilateral salpingoophrectomy.

Intraoperative complication was reported in two patients (7.4%), whereas small intestinal and urinary bladder injury was reported equally in one patient. The 30-day postoperative complications were reported in four cases. The most common was wound-related, including either seroma, wound infection, or burst abdomen. Recurrence was reported in one patient with serous type after approximately 26 months. Resection of the recurrent mass was performed where pathological assessment revealed a recurrent ovarian borderline serous tumor. One patient succumbed to pulmonary embolism in the first month after her surgery.

The most common pathological type in our cohort was the mucinous borderline type reported in 14 patients (51.9%), followed by the serous type reported in 11 patients (40.7%) and the seromucinous type in 1 patient only. The microinvasive component was reported in six patients (22.2%); one of these patients had bilateral synchronous borderline line serous tumor on one side and low-grade serous carcinoma on top of borderline serous tumor on the contralateral side.

We compared the parameters of patients diagnosed with mucinous borderline type with those of serous and seromucinous types (Table 3). Patients with the mucinous borderline type were significantly younger (40.083 ± 18.47 vs. 53.73 ± 11.91 years, *p* = 0.028). Patients with serous and seromucinous types presented more with pelviabdominal mass than those with the mucinous type who presented with vague abdominal pain. Interestingly, Cancer Antigen 125 levels were significantly higher in mucinous than serous and seromucinous types [67(16–304) vs. 20(6–294.6) U/ml, *p* = 0.027]. On the other hand, the radiological tumor size of serous and seromucinous types was larger than that of the mucinous type [23(19–31) cm vs. 8(5–20) cm, *p* = 0.001]. Over a median follow-up period of 58.66 (54.16–63.16) months, only one postoperative mortality was reported, while no recurrence events were reported.

**Table 3:** Sociodemographic and clinicopathological characteristic distribution according to the pathology of the studied patients.

	Mucinous	Seromucinous and serous	Test of significance
Age at diagnosis (years)	40.083 ± 18.47	53.73 ± 11.91	*p* = 0.028*
Body mass index (kg/m^2^)	27.81 ± 5.82	33.95 ± 7.81	*p* = 0.084
Most common presentation			
Pain	8 (80)	2 (16.7)	*p* = 0.018*
Mass	1 (10)	8 (66.7)	
Gastrointestinal symptoms	0	1 (8.3)	
Abnormal uterine bleeding	1 (10)	1 (8.3)	
CA 125	67 (16–304)	20 (6–294.6)	*p* = 0.027*
Tumor side			
Right	3 (25)	8 (53.3)	*p* = 0.05
Left	4 (33.3)	7 (46.7)	
Bilateral	5 (41.7)	0	
CT mass size (cm)	8 (5–20)	23 (19–31)	*p* = 0.001*
Blood transfusion			
No	11 (91.7)	14 (93.3)	*p* = 1.0
Yes	1 (8.3)	1 (6.7)	
Type of intraoperative complications			
Urinary bladder injury	0	1	
Small intestinal injury	1	0	*p* = 1.0
30-day postoperative complications	2	2	*p* = 0.84
30-day mortality	0	1	*p* = 0.32
Microinvasive component	3	3	*p* = 0.76

CA 125, Cancer Antigen 125; CT, computed tomography.

* Statistically significant value when *p* is less than 0.05.

## Discussion

BOTs represent a dilemma whether in preoperative diagnosis based on radiological and clinical signs and symptoms or in frozen section diagnosis during the operation. We discuss our experience as a major oncology hospital in Egypt in managing such cases.

BOTs are reported to be diagnosed usually during the women's reproductive age, with about 30% of cases diagnosed before the age of 40 years (5, 6). The mean age of patients in our study group was 47.67 ± 16.39 years, and the patients with borderline mucinous type were significantly younger than patients diagnosed with borderline serous and seromucinous types (40.083 ± 18.47 vs. 53.73 ± 11.91 years, *p* = 0.028).

Most of our patients (86.4%) were diagnosed with vague abdominal symptoms, mainly abdominal pain and mass. Several authors reported the same findings in diagnosing BOTs (15, 16). A study conducted in Norway revealed that nearly 75% of patients with BOTs had at least a single symptom at the time of diagnosis. Others reported that more than 80% of women with BOTs have variable abdominal symptoms whether abdominal pain, discomfort, distention, or other gynecological and urological symptoms (17–20).

CA-125 had been widely used in the preoperative assessment of ovarian tumors especially in ovarian cancer as a marker that aids diagnosis and gives an indicator of prognosis and response to chemotherapy (21). The use of CA 125 in BOTs is controversial, and several studies reported that it should not be used as a diagnostic tool in BOTs (22, 23). In our study, we reported a median CA 125 level of 33 U/ml, and it was not significantly elevated in most of our patients. Lenhard et al. also reported a median CA 125 level of 34.7 U/ml in a single-center retrospective study, which is nearly the same value reported in our study (24). Another study reported relatively lower CA 125 values in BOT compared to ovarian cancer (25). While few studies reported relatively higher CA 125 levels in borderline serous than in mucinous subtypes (26, 27), we reported significantly higher CA 125 levels in borderline mucinous than serous and seromucinous types.

BOTs, as reported in the literature, are usually diagnosed in an early stage (7). Twenty-two patients in our study, representing 81.4%, were diagnosed with unilateral tumors, while only 5 patients had a bilateral tumor at the time of the diagnosis. Unilateral cystectomy or salpingo-oophorectomy with fertility preservation is now considered the ideal line of treatment in such cases; however, a higher recurrence rate was documented with unilateral cystectomy without affecting the oncological outcome (8–11). In our study, there is heterogeneity in the surgical management of such cases, and this may be attributed to some factors.

The first factor is that, in Egypt, we have a low median age of first marriage of 20.8 years, as reported by UNICEF, compared to western countries, so most of our patients were already married with offspring at the time of surgery, so fertility-sparing surgery was not the choice in such patients. Nineteen patients, representing 70.3% of our study group, had a total abdominal hysterectomy and bilateral salpingo-oophorectomy.

The second factor is that lymphadenectomy was done in eight patients based on clinical and radiological suspicion of ovarian carcinoma instead of BOTs.

The third factor is that we did not use the frozen section examination in all of our patients, and also the frozen section examination was not conclusive in all cases as 8 patients out of 13 (61.5%) were found to be the same diagnosis in paraffin sections as the frozen sections.

According to the WHO classification, the serous borderline tumor represents about 50% of diagnosed cases, followed by mucinous type, which represents about 40% of diagnosed cases. At the final pathology in our study, the most common type was the mucinous borderline (51.9%), followed by the serous type (40.7%), while the seromucinous type was only recorded in one patient. We also reported microinvasion in six patients (22%) in our study. Although the presence of a microinvasive component was reported to be an indicator of a higher recurrence rate (28), other studies denied the association between the presence of microinvasion and the recurrence or survival rate (29, 30). We did not report any recurrences associated with the presence of microinvasion in our study.

Interestingly the radiological tumor size whether by ultrasonography or MRI was significantly larger in serous and seromucinous types than mucinous type in our study 23(19–31) cm vs. 8(5–20) cm, *p* = 0.001), although it is reported in several studies that the mucinous tumors usually tend to be larger in comparison to serous tumors (31–33).

The recurrence rate in BOTs is relatively low and ranges from 0% to 25%, with a higher risk in patients who underwent a unilateral or bilateral cystectomy (3, 8, 34–36). In our study, we did not report any recurrence in a median follow-up period of 58.66 months, but most of our patients were not treated with fertility-preserving surgery as in other studies, and also the relatively small sample size might have an effect on the recurrence rates.

Surgery is considered the main line of treatment for borderline ovarian tumors. Treatment guidelines recommend tailoring the surgical decision according to the histologic and clinical features of the tumor and the age of the patient. Fertility-sparing surgery is a valid option for young females with BOTs, while total hysterectomy and bilateral salpingoophrectomy are reserved for menopausal females. The most common risk factors for relapse or recurrence are ovarian cystectomy and incomplete staging. According to the NCCN guidelines, patients who had incomplete staging or surgery should be managed depending on fertility desire and the presence of invasive implants, where patients who desire to preserve their fertility could be treated by fertility-preserving surgery and resection of any residual disease. The NCCN guideline recommends tailoring the decision of lymphadenectomy on a case-by-case basis in view of the available evidence from the literature about no improved survival after lymphadenectomy and omentectomy for BOTs. In the present study, conservative surgery was performed in 29.4% of the patients, while more aggressive approaches including omentectomy and/or lymphadenectomy were performed in 55.5% of them.

The present study highlighted the management of borderline ovarian cancer in a tertiary center in a developing country. We believe that this study is crucial to arouse concerns about the urgent need to unify the management of this type of tumor among the gyneoncologists in developing countries. However, it is worth mentioning that this study had some limitations, including the retrospective design, the relatively small sample size, and the heterogeneity of the surgical management as not all the patients were operated by gynecologic oncologists and most of our patients did not have a fertility-sparing surgery.

Although most guidelines consider complete surgical staging as the standard of care in the management of BOTs, a large proportion of the patients is incompletely staged (37). The main concern about incomplete staging is that it could hinder the detection of advanced diseases that could be surgically resected or confirm the need for adjuvant therapy. In their series, Romeo et al. reported that 10.9% of relapse in all of them was staged as FIGO stage I after incomplete staging (37). In the present study, no recurrence events were reported in patients who underwent conservative surgery.

Interestingly, the benefit of restaging surgery after the incomplete staging is still debatable. In their study, Lecointre et al. highlighted the main indications of restaging surgery as cystectomy in mucinous tumor or serous tumor with a micropapillary component, incomplete peritoneal exploration, a defective surgical technique that led to seeding, and peritoneal lesions after the primary surgery in the case of residual gross (38).

Borderline ovarian tumors still represent a dilemma either in diagnosis or management. A frozen section examination could help to reach a preliminary diagnosis. Total abdominal hysterectomy and bilateral salpingo-oophorectomy are the cornerstone of surgical management. However, fertility-sparing surgery could be a valid option for women desiring fertility. Future studies are awaited to address the incidence of recurrence and its risk factors to guide the best management protocol.

## Data Availability

The raw data supporting the conclusions of this article will be made available by the authors, without undue reservation.
